# Supplementary material for: High throughput single-cell detection of multiplex CRISPR-edited gene modifications
Source: Genome Biol. 2020 Oct 20;21:266. doi: 10.1186/s13059-020-02174-1 (PMC7574538; doi:10.1186/s13059-020-02174-1)
Supplement: Supplementary file 1 — Figure S1. Single cell DNA-seq of singly-edited Ba/F3 cell lines. QC metrics of single-cell DNA-seq analysis by Tapestri including number of sequencing reads and targeted amplicon coverage, analysis of percent modified reads across on and off-targets and zygosity analysis of on-targets. Figure S2. Single cell qPCR-based detection of sgRNAs. Analysis of number and co-occurrence of sgRNAs in single Ba/F3 cells, as assessed via qPCR-based detection. (DOCX 689 kb) [file 13059_2020_2174_MOESM1_ESM.docx]

**Additional File 1: Figure S1.**

**
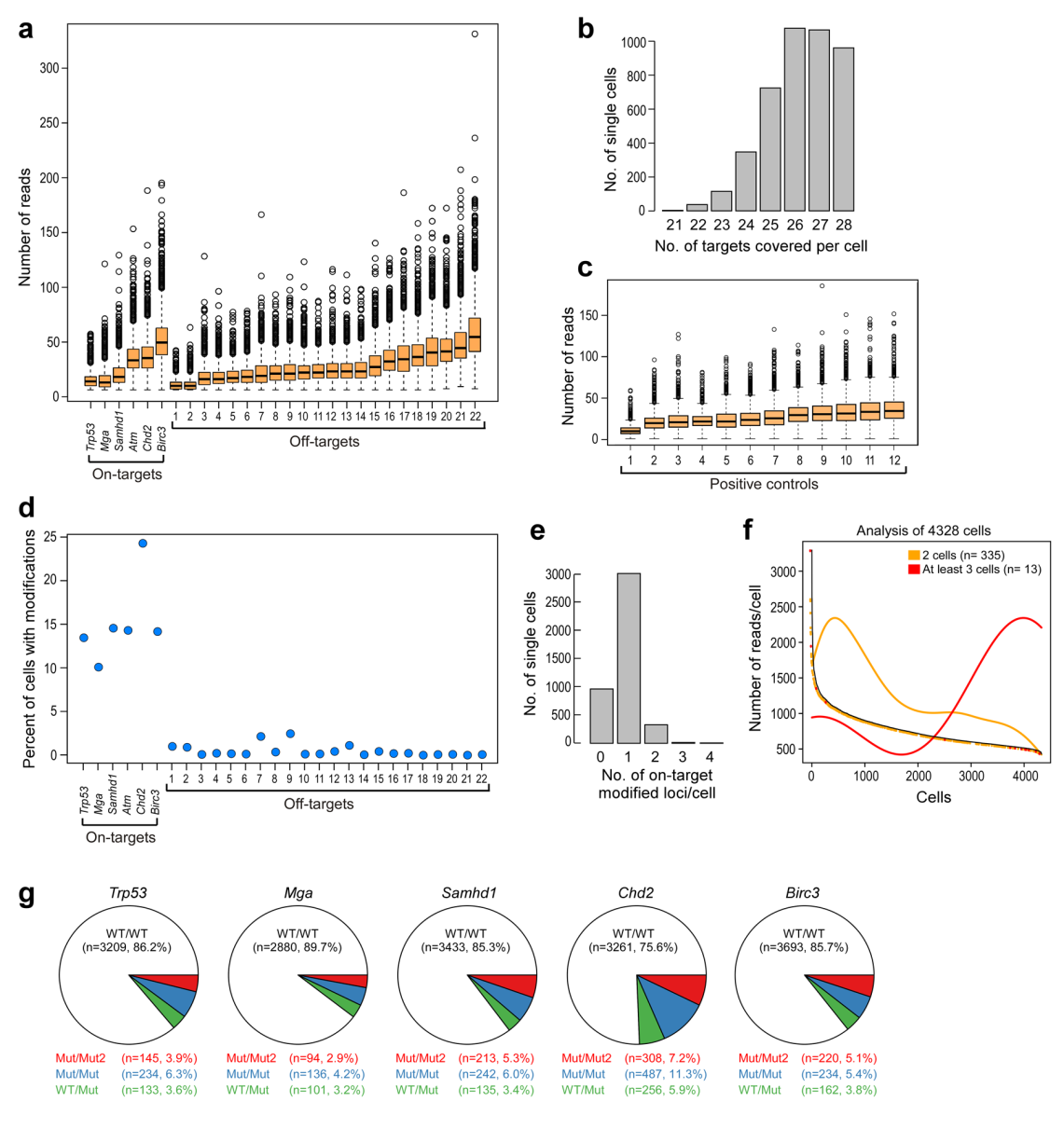
**

**Fig S1. (a)** Amplicon coverage per cell across all tested on and off-targets. The median coverage per cell is shown by the thick horizontal line. The box shows the interquartile range, whiskers extend to data ranges 1.5 times the interquartile range, and outliers outside of this range are shown with dots. **(b)** Bar diagram reporting number of targets detected in each cell, with sufficient coverage. **(c)** Amplicon coverage per cell across the 12 positive control amplicons. (**d**) Percent cells containing modifications across the 6 on-targets and the 22 off-target regions. **(e)** Bar diagram reporting number of sites carrying modifications across all the 4328 assayed cells. **(f)** Doublets/triplets detection based on cell-barcodes that show editing at more than one target. The black line shows the number of aligned reads per cell-barcode for all 4328 cell-barcodes, and the cell-barcodes are ordered by the number of aligned reads assigned to that cell-barcode with the cell-barcode with the highest number of reads on the left and the cell-barcode with the lowest number of reads on the right. Note that although we excluded cell-barcodes with fewer than 1000 reads from downstream analysis, this plot shows the number of reads aligned to our 28 amplicons (e.g. excluding reads aligned to positive controls). Cells with two edits are marked in orange, and cells with three edits are marked in red. The density distribution across the range of aligned reads per cell-barcode are shown as solid lines in corresponding colors. **(g)** Pie chart referring to heterozygous/homozygous subpopulations of gene edits within the *Trp53, Mga, Samhd1, Birc3* and *Chd2* on-target loci. Numbers and percentage read counts for each sub-group are shown.

**Figure S2.**


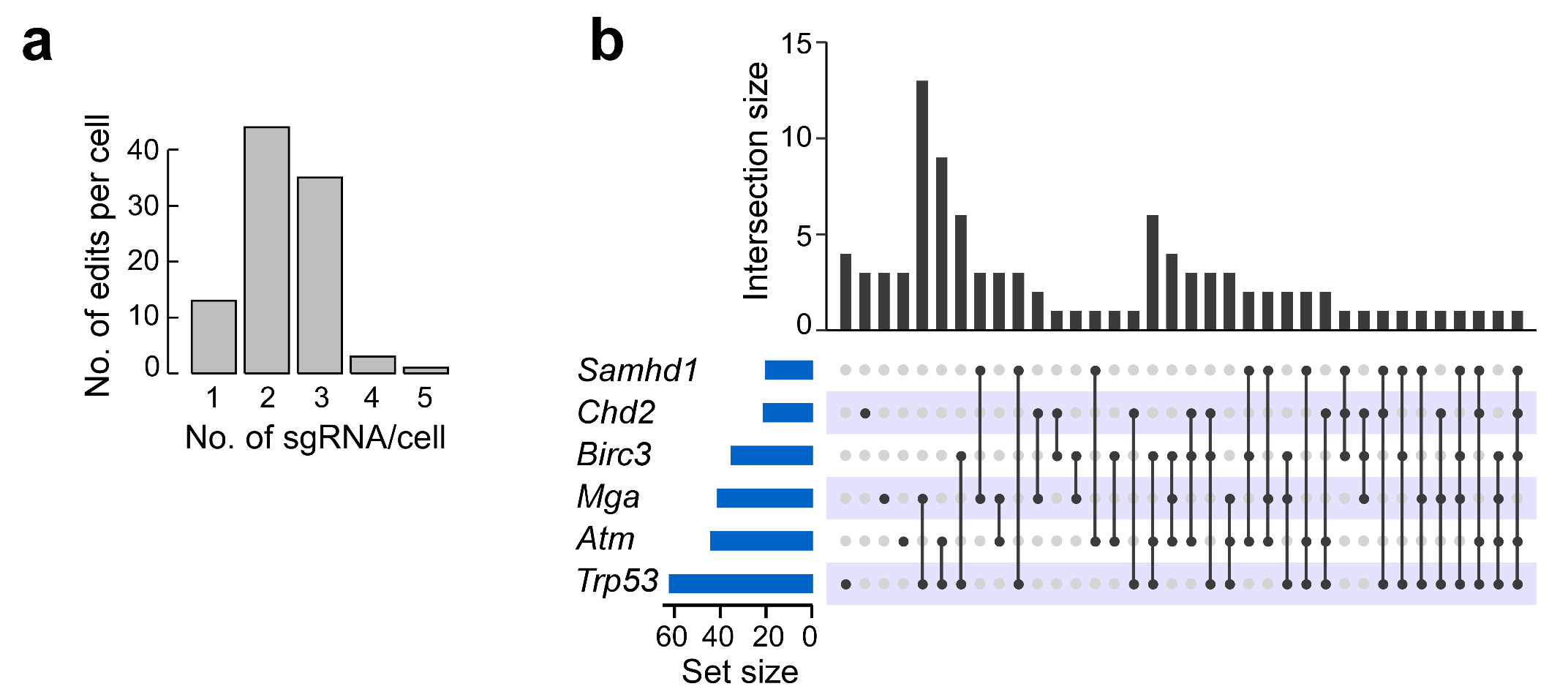


**Fig S2. (a)** Barplot reporting number of sgRNAs detected by single-cell qPCR in 96 cells. **(b)** Histogram showing sgRNA expression across the 96 cells assayed by single-cell qPCR. Set size refers to the number of cells for each of the six targets. Intersection Size refers to the number of cells for each sgRNA combination.
